# Editorial: Neuroinflammation and gut-brain axis: role of glia cells

**DOI:** 10.3389/fnins.2025.1618286

**Published:** 2025-06-06

**Authors:** Deiziane V. S. Costa, Danielle S. Macedo, Camila N. C. Lima

**Affiliations:** ^1^Division of Infectious Diseases and International Health, University of Virginia, Charlottesville, VA, United States; ^2^Department of Physiology and Pharmacology, Faculty of Medicine, Neuropsychopharmacology and Tranlational Psychiatry Laboratory, Drug Research and Development Center, Federal University of Ceara, Fortaleza, Brazil

**Keywords:** gut-brain axis, microbiota, glia, dementia, neurodegeneration

Gut inflammation has been bidirectionally associated with neurological and psychiatric conditions in humans, supporting a potential role of the gut-brain axis (Heston et al., 2023; Bisgaard et al., 2023a,b; Rajkovaca Latic et al., 2024). Cognitive impairment, anxiety, and depressive-like behavioral phenotypes have also been reported to occur following recovery from gut inflammation in a preclinical mouse model (Zhang et al., 2022; Lee et al., 2024), allowing mechanistic studies and development of potential therapeutics. In dementia patients, increased levels of microbe-derived molecules such as p-cresol, iso-butyric acid and iso-valeric acid have been detected in fecal samples (Saji et al., 2020), which supports the contribution of gut-derived molecules to this neurological condition.

**Figure 1:**
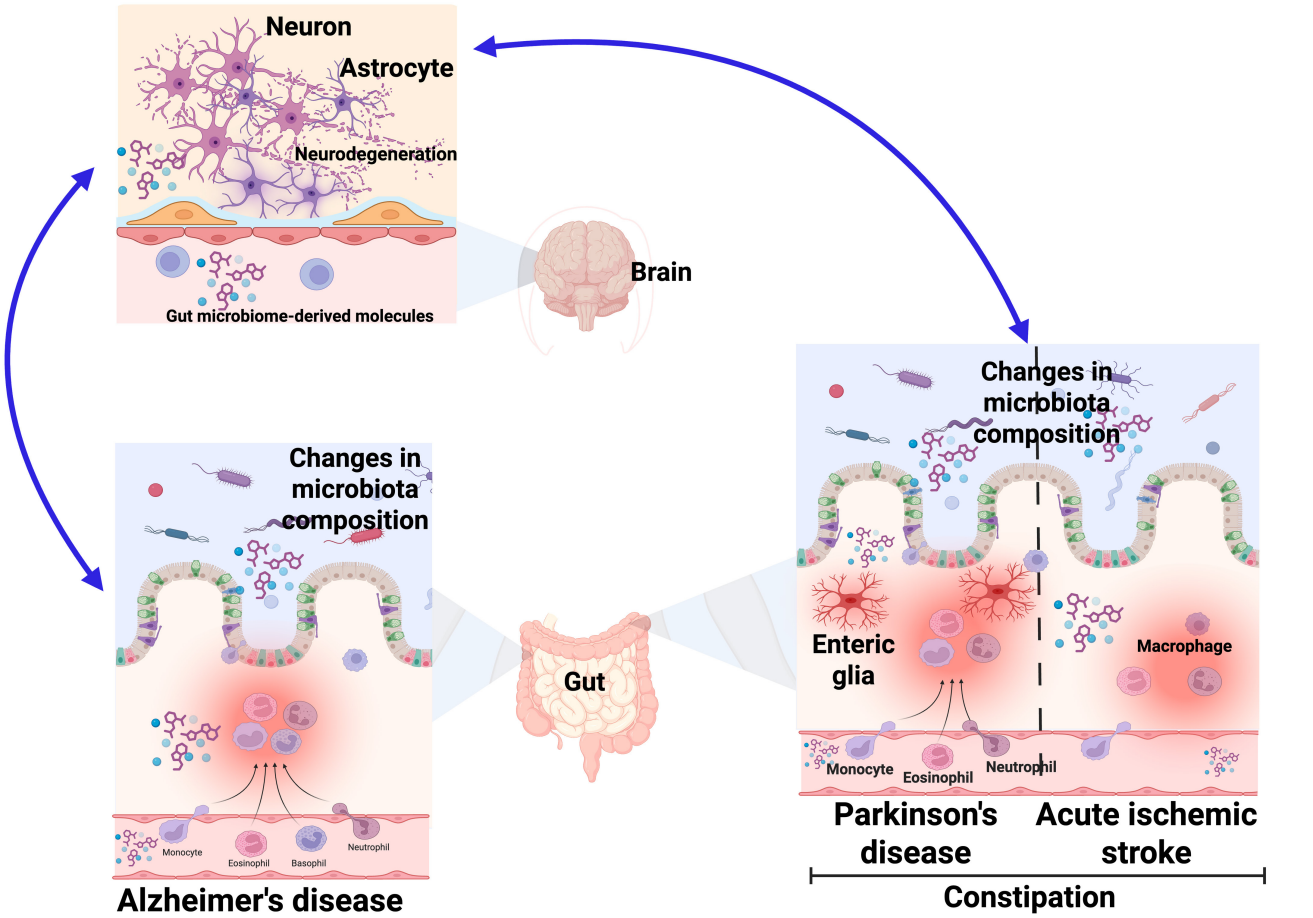
Impact of the gut-brain axis on neurodegenerative diseases. Overview of the included studies on the Research Topic Gut-Brain Axis: Role of Glial Cells.

Dementia is a general term for loss of memory and other thinking abilities severe enough to interfere with daily life. Stroke, Alzheimer's disease (AD) and Parkinson's disease (PD) are common causes of dementia (Livingston et al., 2024). The four papers collected for our special issues, which included three reviews and one original article, discussed about how gut microbiota and/or enteric glia contributes to acute ischemic stroke (AIS), AD, and PD (Figure 1).

Deng et al. performed an epidemiologic study of 499 acute ischemic stroke (AIS) patients in combination with preclinical model studies in the Bama miniature (BM) pig model to investigate the contribution of gut microbiota-derived metabolites in AIS. A phenomenon of delayed bowel movements was observed in large hemispheric infarction (LHI) patients, which may be induced by a strike to the gut-brain axis. ^1^H NMR-based metabolomics was also employed to reveal a global metabolic profile in the plasma of AIS patients. Subsequently, a Bama miniature (BM) pig model with LHI was successfully established and employed to investigate alterations in intestinal barrier integrity, the gut microbial community, and microbiota-derived metabolites. These findings highlight the contribution of the microbiome-gut-brain axis in both clinical AIS patients and LHI BM pigs.

Alves et al. and Yan et al. provided an up-to-date review with a focus on AD. Alves et al. highlighted the potential of *Drosophila* as a robust model to investigate the association between the fly gut microbiota and the human microbiota, a connection that helps uncover the mechanisms linking bacterial balance to AD progression and inform future therapeutics. Yan et al. reviewed recent evidence on the role of acupuncture to modulate the gut microbiota as a therapy for AD and found that the evidence suggests that acupuncture combined with modulation of the gut microbiota may be beneficial in the treatment of AD in the future.

Finally, Thomasi et al. performed an elegant review on the potential role of enteric glia in PD. The authors focused on generating potential hypotheses on how enteric glial dysfunction may be connected to the development and/or progression of PD. Based on clinical data and animal models of PD in combination with other models of gut inflammation, the researchers explained how enteric glia and microbial molecules can affect each other, and how this interaction can impact gut dynamics, and the role of enteric glia in enteric neurotransmission, dysmotility, and visceral hypersensitivity. All of these aspects, at different stages of the disease, may be related to PD.

All these studies have provided insights into potential mechanisms related to gut-derived molecules, specifically microbiota and/or enteric glia, that could be targeted to prevent the progression of brain neuroinflammation associated with AD, PD and stroke.
